# Overcoming drug resistance by targeting protein homeostasis in multiple myeloma

**DOI:** 10.20517/cdr.2021.93

**Published:** 2021-12-02

**Authors:** Maria Moscvin, Matthew Ho, Giada Bianchi

**Affiliations:** 1 Department of Medicine, Division of Hematology, Brigham and Women’s Hospital, Boston, MA 02115, USA; 2 Department of Medicine, Mayo Clinic, Rochester, MN 240010, USA

**Keywords:** Multiple myeloma, drug resistance, proteasome inhibitors, immunomodulatory drugs, proteostasis, endoplasmic reticulum stress, unfolded protein response

## Abstract

Multiple myeloma (MM) is a plasma cell disorder typically characterized by abundant synthesis of clonal immunoglobulin or free light chains. Although incurable, a deeper understanding of MM pathobiology has fueled major therapeutical advances over the past two decades, significantly improving patient outcomes. Proteasome inhibitors, immunomodulatory drugs, and monoclonal antibodies are among the most effective anti-MM drugs, targeting not only the cancerous cells, but also the bone marrow microenvironment. However, *de novo* resistance has been reported, and acquired resistance is inevitable for most patients over time, leading to relapsed/refractory disease and poor outcomes. Sustained protein synthesis coupled with impaired/insufficient proteolytic mechanisms makes MM cells exquisitely sensitive to perturbations in protein homeostasis, offering us the opportunity to target this intrinsic vulnerability for therapeutic purposes. This review highlights the scientific rationale for the clinical use of FDA-approved and investigational agents targeting protein homeostasis in MM.

## INTRODUCTION

Multiple myeloma (MM) is a clonal proliferation of malignant plasma cells, the product of the terminal differentiation of B cells. Clinically, MM is suspected based on the presence of laboratory abnormalities such as hypercalcemia, anemia, renal failure, or radiological evidence of lytic bone disease^[1]^.

The clinical use of molecularly targeted agents, including bortezomib, the first in class proteasome inhibitor (PI), immunomodulatory drugs (IMiDs) like thalidomide, lenalidomide and pomalidomide, and monoclonal antibodies targeting CD38 and SLAMF7 has dramatically impacted the life expectancy of MM patients. All these drugs have successfully passed regulatory approvals and are used in patients with newly diagnosed and/or relapsed, or refractory disease, in all stages of treatment, contributing to prolonging median overall survival to 7-8 years.

Despite an improved understanding of the pathobiology of myeloma and a significant drug-development effort in the past 2 decades, MM remains incurable and therapeutic resistance represents a major clinical concern. Even in patients whose disease initially responds to treatment, the acquisition of resistance to chemo-immunotherapy over time is a common phenomenon. The molecular mechanisms underlying such acquired resistance are only partially understood, thus limiting therapeutic success.

One pathognomonic feature of myeloma cells is the exuberant production of clonal, intact immunoglobulin and/or free light chains. MM cells contain a well-developed endoplasmic reticulum (ER) and Golgi apparatus, tailored for such sustained protein synthesis and secretion effort^[2,3]^. By virtue of their nature as factories of protein production, MM cells are especially prone to improper protein folding of nascent Ig and baseline proteotoxic stress^[4,5]^. Drugs that further dysregulate protein quality control and proteostasis have shown to be specifically toxic against MM by exacerbating proteotoxic stress and causing apoptosis.

In recent years, extensive research has focused on understanding the role that protein homeostasis (proteostasis) plays in supporting efficient protein synthesis, folding, secretion, and degradation and on the identification of putative molecular targets within this complex network^[6–9]^.

In this review, we will outline the current understanding of protein metabolism and homeostasis in MM and the rationale for translating laboratory discovery in proteostasis into patient-focused therapies.

## BIOLOGICAL RATIONALE FOR TARGETING THE PROTEOSTASIS NETWORK IN MM

Eukaryotic cells maintain a balanced proteome through the function of the proteostasis network, composed of a large number of proteins regulating four interlinked pathways: protein synthesis, folding, secretion, and degradation^[5]^.

**Figure 1.**
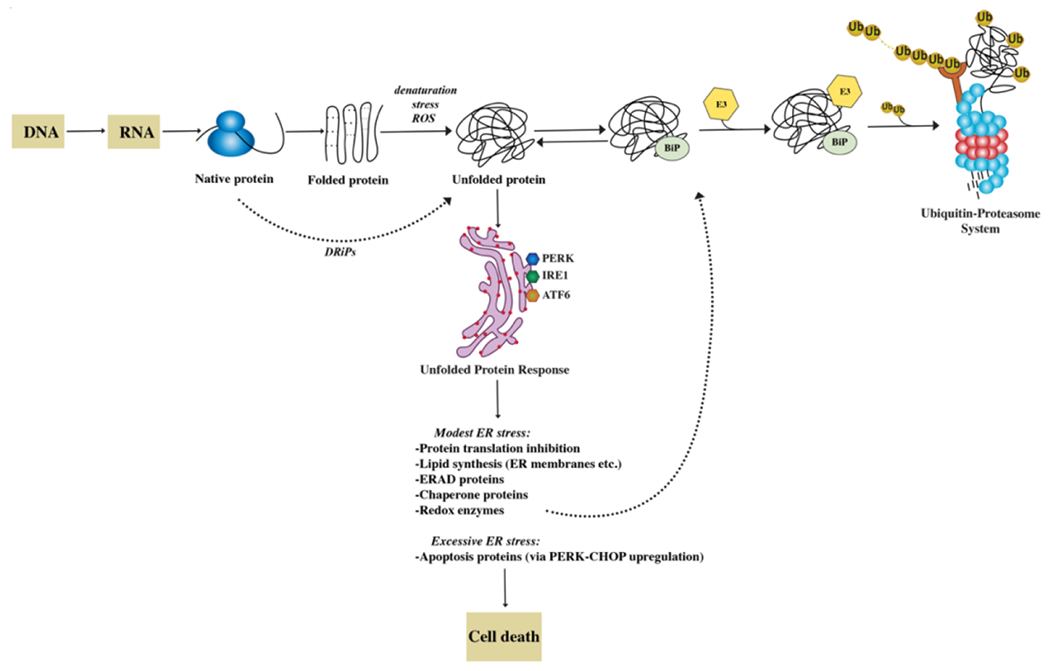
The proteostasis network. The folding of newly synthesized proteins is a complex mechanism that involves multiple steps. ~30% of nascent proteins, named DRiPs, have an inherent inefficiency of protein folding and undergo degradation within minutes from synthesis. Most proteins achieve a functional folded state. However, many are the causes that trigger spontaneous unfolding. These changes in conformation are recognized by the cell’s protein quality control machinery with activation of an unfolded protein response (UPR). The three branches of the UPR (PERK, IRE, ATF6) help restore protein homeostasis partially by increasing the synthesis of chaperone proteins. By association with exposed hydrophobic domains, chaperones like BiP (GRP78), favor refolding. Alternatively, they can facilitate the recognition of abnormal proteins, leading to their ubiquitylation by E3 and their degradation through the proteasome. If the ER stress cannot be mitigated and homeostasis cannot be reestablished, UPR induces cell death.

After synthesis, secreted proteins undergo an intricate process of folding involving sequential steps of post-translational modifications, such as glycosylation and disulphide bond formation, taking place in the ER^[10]^. Molecular chaperones assist client proteins during folding, exerting a stringent quality control that allows only proteins that reach a native conformation to progress along the secretory pathway [Figure 1].

Nascent proteins that fail to do so and remain misfolded are diverted to proteasome-mediated degradation in a process called ER-associated degradation (ERAD). Thus, the proteostasis network also ensures that superfluous and misfolded proteins are removed by the ubiquitin-proteasome system (UPS). Alternative proteolytic mechanisms cooperate with the UPS in guaranteeing proteostasis, such as the macroautophagy (autophagy-lysosome) system and the aggresome pathway^[5,11]^. In fact, the accumulation of misfolded proteins is sensed as toxic by cells and highly conserved adaptive responses, such as the unfolded protein response (UPR) and the heat shock response (HSR), exist with the goal of restoring protein homeostasis.

The UPR is a tripartite protein homeostasis mechanism triggered by the accumulation of misfolded proteins within the ER (ER stress). The ultimate goal of the UPR is to reduce protein toxicity by decreasing total protein synthesis while selectively upregulating chaperone transcription and translation^[11]^. However, if homeostasis cannot be restored, the UPR activates a terminal pathway, triggering C/EBP homologous protein (CHOP) and GADD34 transcription and ultimately apoptosis. The HSR similarly induces upregulation of chaperons via the master-regulator heat shock factor 1 (HSF1) to counteract protein misfolding^[12,13]^.

The UPS plays a central role in maintaining protein homeostasis as it is the main proteolytic mechanism responsible for the degradation of misfolded proteins. Clearance of these specimens is critical to both avoid toxicity and recycle ubiquitin and amino acids.

Studies have shown that treatment of MM cells with PI perturbs proteostasis adaptive responses, impairs DNA repair, and eventually triggers apoptosis. Indeed, increased proteasomal workload (polyubiquitinated proteins), decreased proteasomal capacity, or a combination of both is a key determinant of PI sensitivity in MM^[14]^. Consistently, drugs that increase proteasome cargo such as ER stressors and heat shock protein inhibitors synergize with Pis^[15]^. Further works assessing combination therapies targeting one or more of these pathways in MM are currently ongoing.

## THE UBIQUITIN-PROTEASOME SYSTEM

Protein translation and folding are imperfect mechanisms, and it is estimated that up to 30% of nascent proteins have an intrinsic inability to achieve stably folded conformations and never reach a functional state. These unstable proteins are termed defective ribosomal products (DRiPs)^[16]^. It is thought that such faulty protein species are even more abundant in highly secretory, malignant cells such as myeloma PC that synthesize extensive amounts of immunoglobulins. DRiPs contribute to proteotoxic stress by inducing the UPR and potentially overwhelming the UPS and ERAD, resulting in the accumulation of polyubiquitinated proteins^[17,18]^.

The UPS is primarily responsible for the degradation of misfolded polypeptides, accounting for over 80% of total proteolysis^[19,20]^. Secreted and membrane proteins are used by cells to communicate with their environment and account for approximately 30% of the cellular proteome^[10]^. Before they are inserted into cellular membranes or released into the extracellular space, they must achieve their native conformation in the ER lumen. If they fail to do so, they are retro-translocated via the Sec61 translocon from the ER to the cytosol for ubiquitination and degradation by the proteasome in a cellular pathway termed ERAD^[21]^.

The proteasome is an ATP-dependent, multi-catalytic protease mediating degradation of senescent and/or misfolded proteins that are generally tagged for degradation via K48-linked polyubiquitin chains^[19]^. Protein ubiquitination is controlled by a three-enzyme cascade involving E1 (activating), E2 (conjugating), and E3 (ligating) enzymes. On the other hand, the large family of deubiquitinating enzymes (DUBs) remove ubiquitin, critically contributing to recycling ubiquitin and maintaining a steady-state pool of free ubiquitin^[22,23]^. Polyubiquitinated proteins are delivered to the 26S proteasome, a barrel-like complex composed of a 20S catalytic core, associated with 19S regulatory caps^[24]^. The regulatory subunits mediate recognition, binding, unfolding, and facilitate engagement of targeted proteins with the 20S catalytic core. Together with DUBs (e.g., UCH37, USP14), they also engage in the removal of polyubiquitin chains, which would otherwise impede transferring the cargo proteins through the tight pore created by the 19S cap on the 2 sides of the 20S core^[25]^. The 20S core contains β1, β2, β5 main catalytic subunits, which are responsible for caspase-like, trypsin-like, and chymotrypsin-like activities, respectively^[24]^.

In addition to the constitutive proteasome, immune cells can be equipped with immunoproteasome, typically in the setting of infection or pro-inflammatory stimuli^[26]^. The immunoproteasome subunits β1i (LMP2), β2i (MECL-1), and β5i (LMP7) replace the constitutive catalytic subunits^[26,27]^. It has been previously shown that MM cells are equipped with large amounts of immunoproteasome, and selective inhibition of these catalytic activities is an appealing therapeutic strategy for MM and potentially other hematologic malignancies^[28,29]^.

### Proteasome inhibitors

**Figure 2.**
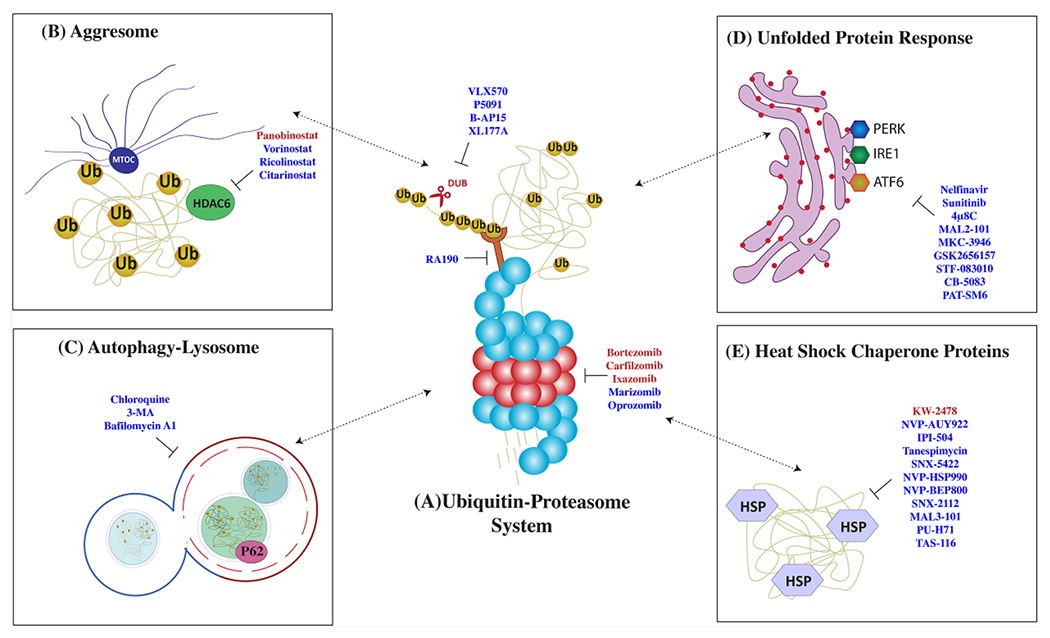
Therapies targeting protein control pathways in multiple myeloma (MM). MM cells are highly dependent on a strictly regulated network of protein quality control pathways such as (A) the ubiquitin-proteasome system (UPS), (B) aggresome formation, (C) autophagy, (D) unfolded protein response, and (E) the heat shock response. Drugs that target these pathways are listed here: FDA-approved drugs (in red) and experimental drugs (in blue).

PIs were initially developed in the mid-1990s as a research tool to investigate proteasome-mediated proteolysis mechanisms^[30,31]^. The clinical use of PI has radically changed the natural history of MM and now, along with immunomodulatory agents and monoclonal antibodies, form the backbone of MM treatment. These agents paved the way for therapies targeting protein homeostasis in cancer. The Pis bortezomib, carfilzomib, ixazomib, and oprozomib show inhibitory activity mainly against the β5 chymotrypsin-like subunit, while marizomib targets all three β-subunits [Figure 2A]^[32,33]^.

Although the clinical use of PI was predicted to have significant toxic effects related to the ubiquitous expression of the proteasome, Pis are generally well tolerated, with distinct toxicities across different generations consisting of hematologic, gastrointestinal toxicities, and peripheral neuropathy, usually readily manageable^[34]^.

The proposed molecular mechanisms of bortezomib anti-MM activity imply pleiotropic levels of actions, including direct actions on both the tumor cells and the BM microenvironment. In the context of the BM microenvironment, bortezomib modifies the cytokine milieu, has anti-angiogenic activity, impairs stroma-tumor crosstalk, and induces apoptosis of osteoclasts while supporting osteoblastogenesis^[2,35,36]^.

The proposed mechanistic explanations for the direct anti-myeloma plasma cell effect include inhibition of pro-survival via NF-κB pathway regulation, impairment of DNA damage response, apoptosis via both caspase 8 and 9 cleavage and regulation of various members of the B-cell lymphoma protein family^[37,38]^. Induction of HSR, UPR, c-Jun NH2-Terminal Kinase, and TP53 are additional hypothesized mechanisms of PI action^[39]^.

Importantly, despite initial fear of intolerable side effects, considering the ubiquitous and essential expression of the proteasome, MM are exquisitely sensitive to PI, with several lines of evidence suggesting that this can be attributed to baseline proteotoxicity in MM plasma cells^[2,40,41]^. In fact, both Ig synthesis and retention correlate with apoptotic sensitivity to PI, and manipulating Ig synthesis alters sensitivity^[14]^. The abundant production of Ig, paired with insufficient proteostasis mechanisms, leads to baseline proteotoxic stress that can be further exacerbated by PI, resulting in apoptosis. Increasing protein misfolding via ER stressors results in increased sensitivity to PI, while augmenting proteasome activity or inducing alternative proteolytic pathways mediates PI resistance^[14]^. These preclinical data suggest potential mechanisms of acquired resistance to PI.

Resistance to PI is ultimately inevitable in most MM patients, leading to refractory disease and negatively impacting outcomes. Research efforts have focused on identifying the molecular mechanisms of PI resistance to develop novel therapies able to overcome it. We and others have recently shown that targeting the compensatory proteasome stress response (PSR) is of therapeutic utility in MM and can overcome acquired or de novo PI resistance^[42]^. The transcription factor Nuclear Factor Erythroid 2 Like 1 (NFE2L1 or NRF1) is the master regulator of the PSR^[42]^. Under homeostatic conditions, NRF1 is continuously translated, inserted into the ER, glycosylated, retro-translocated to the cytosol, and targeted for proteasomal degradation via ERAD. Though, when proteasome activity is partially inhibited, NRF1 is deglycosylated by N-glycanase 1 (NGL1) and cleaved by the aspartic protease DNA-damage inducible 1 homolog 2 (DDI2). NRF1 in its active form translocates to the nucleus and dimerizes with small MAF proteins, leading to a complex transcriptional program that includes the biogenesis of new proteasome subunits^[43–47]^. Recent studies show that the genetic or pharmacologic blockade of NGL1 increases sensitivity to PI-mediated cytotoxicity, suggesting its therapeutic potential^[42]^. Recently, similar results were obtained via knock out of DDI2 or NRF1 directly^[48]^.

A different strategy to increase PI activity has focused on developing next-generation PI characterized by increased potency, irreversible catalytic activity blockade, and/or broader catalytic subunits inhibition.

The first-in-class PI bortezomib is a peptide boronic acid that reversibly inhibits the β5 subunit. It is approved for administration via intravenous (i.v.) or subcutaneous (s.c.) injection^[7]^. By contrast, carfilzomib is an epoxyketone, a second-generation agent that irreversibly inhibits the β5 subunit. Preclinical studies showed that carfilzomib has a higher potency than bortezomib, and clinical trials showed durable responses to a single agent and combination therapy in patients relapsed/refractory to bortezomib and lenalidomide treatment^[32,33,49]^. These positive results led to its FDA approval in combination with lenalidomide and dexamethasone (Rd), as the second line of treatment in MM, by i.v. administration. Interestingly, the pattern of side effects of carfilzomib is quite distinct from bortezomib. While the latter can cause cardiovascular side effects, including tachyarrhythmia, hypertension, and systolic heart failure, the latter is often responsible for sensory peripheral neuropathy.

Ixazomib and oprozomib are orally bioavailable Pis chemically related to bortezomib and carfilzomib, respectively. The former is currently FDA approved for the treatment of RRMM, while the latter showed promising activity as a single agent and in combination with IMiDs in RRMM^[50,51]^.

Finally, the irreversible agent marizomib has a unique β-lactone warhead, and, unlike all other clinically available PIs, it inhibits all three catalytic subunits within the 20S core^[52]^. Recent preclinical studies show potent activity of marizomib even in bortezomib-resistant MM cells, suggesting that broader blockade of proteasome subunits may increase effectiveness^[53,54]^. However, this potent and universal proteasomal subunits inhibition may result in a narrowing of the therapeutic index. Indeed, early phase clinical trials show that the potent inhibition of proteasome subunits was similarly accompanied by renal and central nervous system toxicity, hampering clinical development of this derivative in MM^[54]^.

### Deubiquitinating enzymes and ubiquitin receptors inhibitors

Potential molecular targets within the UPS include enzymes involved in ubiquitination (E1, E2, E3) and deubiquitination (DUBs) of proteins destined for proteasomal degradation^[55,56]^. The development of therapeutically effective inhibitors in this area can be challenging considering the numerous members of ubiquitinating and DUBs, their distinct structure, and the potential for systemic toxicities related to the nature of client proteins. Similar to PI, DUB inhibitors induce apoptosis in preclinical MM models preceded by accumulation of polyubiquitinated proteins and in a manner that is independent from the inhibition of the proteasome catalytic activity^[57]^. Therefore, there is a scientific rationale to predict that DUBs would overcome PI resistance by more generically targeting proteostasis. In fact, small molecule compounds P5091, B-AP15, and VLX1570 successfully induced apoptosis in multiple myeloma cell lines and primary cells, including those resistant to bortezomib [Figure 2A]^[58]^.

RPN13 is a polyubiquitin receptor (UbR) within the 19S subunit. Rpn13 binds with high-affinity ubiquitin carboxyl-terminal hydrolase L5 (UCHL37), the deubiquitinating enzyme that helps with ubiquitin (Ub) hydrolysis^[59]^. RPN13 and UCHL37 have been found to be relevant for cell cycle progression *in vitro*, and increased expression of the gene encoding RPN13 (ADRM1) has been reported in MM cells when compared to normal plasma cells^[60]^. Preclinical studies of RA190, a specific, small molecule inhibitor of RPN13, showed robust antitumor activity^[61]^. RA190 decreased the viability of MM cell line and patient-derived MM cells by inducing caspase-dependent apoptosis and UPR. Combination of RA190 with bortezomib, lenalidomide, or pomalidomide induces synergistic anti-MM activity, providing the framework for clinical evaluation of RPN13 inhibitors^[60]^. WL40 is a first in class, RPN13 degrader through proteolysis-targeting chimeric molecule^[62]^. WL40 was synthesized by fusing RA190 with cereblon (CRBN) binding ligand thalidomide, triggering degradation of cellular RPN13. Importantly, WL40 not only decreases the viability of patient MM cells, even those resistant to bortezomib, but prolongs the survival of xenografted human MM models. As predicted, WL40 induces cytotoxicity by activating ER stress response, p53/p21 signaling, and ultimately caspase apoptotic cascade^[62]^.

The first in human UCHL5 inhibitor VLC1570 similarly showed promising and potent anti-MM activity *in vitro*; however, clinical development was halted due to fatal lung toxicity observed in a first in human, phase I study^[63]^.

Based on the pattern of client proteins, USP7 has been proposed as a therapeutic target across numerous, distinct cancers^[64,65]^. P5091, a USP7 inhibitor, showed promising results in overcoming PI-resistance in MM via direct anti-MM activity alone or in combination with anti-MM agents. Similarly, a novel USP7 inhibitor, XL177A, was cytotoxic against MM in preclinical models and appeared to target supporting plasmacytoid dendritic cells to restore anti-MM immunity^[58,66]^. Recently, XL177A showed p53-dependent cytotoxicity against Ewing sarcoma and malignant rhabdoid tumor^[67]^.

### Ubiquitin enzymes

The process of ubiquitin conjugation to target proteins is highly dynamic and involves the regulated, sequential activity of three classes of enzymes: E1, E2, and E3 Ub enzymes^[68]^. Despite the diversity of E2 and E3 ubiquitin enzymes, there are only two E1 ubiquitin-activating enzymes. Thus, inhibiting the E1 ubiquitin-activating enzyme would theoretically block all ubiquitin-dependent pathways in cells. Recently, TAK-243, a potent and selective inhibitor of E1 Ub activating enzyme, suppressed myeloma cell line and primary cells viability through activation of protein kinase RNA-like ER kinase (PERK) arm of the ER stress response pathway, as well as induction of oxidative stress^[69]^. Comparable anti-MM activity was reported in murine myeloma models, supporting a potential clinical use of this strategy in RRMM, although the therapeutic index may not be favorable^[70]^.

Since E3 proteins determine substrate specificity, it is not surprising that over 600 E3 enzymes are encoded by the human genome. These E3 ligases are generally classified into three large families with distinct catalytic domains: really interesting new gene (RING), homology to E6-Ap carboxyl terminus (HECT), and RING-in-between-RING (RBR)^[71,72]^.

The RING class of E3 enzymes acts as a docking site to bring together the targeted substrate designated for degradation with the E2-Ub, thus working as an allosteric activator^[73]^. The HECT and RBR E3 classes catalyze substrate ubiquitination by undergoing a cysteine-dependent transthiolation reaction with E2-Ub, forming a covalent E3-Ub intermediate, and later the Ub moiety is transferred to a lysine on the target substrate^[74]^. The E3 ligase cereblon (CRBN) is the main target for anti-myeloma activity of IMiDs thalidomide, lenalidomide, and pomalidomide^[75]^. Lenalidomide has been shown to bind to the E3 ubiquitin-ligase complex composed of damage-specific DNA binding protein 1 and CRBN, enhancing its activity and facilitating ubiquitination and proteasome-mediated degradation of the Ikaros family of transcription factors^[76,77]^.

Cullin-RING E3 are post-translationally activated by NEDD8 activating enzyme (NAE) in a process called neddylation^[78]^. Therefore, inhibiting neddylation would result in cullin-RING E3 ubiquitin ligase blockade. Inhibitors of NAE have been developed and have been under clinical investigation for a variety of cancer cell types. A Phase I clinical study on pevonedistat (MLN4924), an NAE inhibitor, showed modest activity in lymphoma, but no significant activity in MM^[79]^. Currently, a clinical trial is evaluating the efficacy of the Ixazomib-Pevonedistat combination in RRMM patients.

## AGGRESOME PATHWAY

Targeting alternative proteolytic pathways, such as aggresomes and autophagy, in combination with PI has shown preclinical efficiency by increasing proteotoxic stress^[80]^. HDACs are a group of enzymes responsible for deacetylation of histone and non-histone proteins, resulting in inhibition of gene transcription with contributory effect on critical cellular events such as survival, proliferation, and crosstalk with the surrounding microenvironment^[81]^. HDAC6 coordinates the formation of perinuclear protein aggregates in structures called aggresomes, contributing to maintaining protein homeostasis^[80,82]^. Preclinical studies showed that aggresome formation is a possible mechanism of resistance to PI, and the combination of HDAC inhibitors (HDACi) with PI is synergistic in preclinical MM models^[83]^.

Despite having limited activity as single agents, HDACi have proven to achieve durable responses when in combination with PI and IMiD. Panobinostat, in combination with bortezomib and dexamethasone, was recently approved as third-line therapy in MM patients with prior bortezomib and IMiDs exposure, based on 4-month prolongation of progression-free survival (PFS), near doubling of very good partial response (VGPR), and evidence of response in bortezomib-resistant patients when combined with bortezomib^[84]^. A distinct HDAC inhibitor, vorinostat, was investigated in the phase III, randomized, placebo-controlled, Vantage 008 trial in combination with bortezomib and dexamethasone [Figure 2B]^[85]^. While the experimental group showed a prolonged overall response rate (ORR) and PFS, the clinical relevance of this outcome is not clear.

Frequent and often severe, gastrointestinal and hematologic side effects are the major limitation to the clinical development of HDACi^[86,87]^. Efforts to maintain efficacy and limit toxicities have led to the development of isoform-specific HDACi, focusing on the inhibition of HDAC6 and the aggresome pathway. Two selective HDAC6 inhibitors, ricolinostat (ACY-1215) and citarinostat (ACY-241), are currently being evaluated in clinical studies. A phase 1b trial on ricolinostat in combination with bortezomib/dexamethasone reported a 37% ORR in RRMM, and ricolinostat in combination with lenalidomide/dexamethasone had an ORR of 55% in RRMM^[86]^. Ricolinostat in combination with pomalidomide/dexamethasone is currently evaluated in clinical trials.

BG45, an HDAC3 inhibitor, has also shown promising preclinical results, showing direct and bone marrow microenvironment-mediated anti-MM activity alone and in combination with bortezomib and translation to early phase clinical trial is anticipated soon^[88]^.

## AUTOPHAGY

Autophagy is an evolutionarily conserved mechanism that plays a crucial function in maintaining cellular homeostasis as products of autophagic digestion can be re-utilized in anabolic processes, guaranteeing energy supply^[89]^. Autophagy critically participates in protein homeostasis by sequestering polyUb proteins in autophagic vacuoles that are later degraded upon fusion with lysosomes through an SQSTM1/p62-dependent mechanism^[90]^. While autophagy is typically seen as a pro-survival mechanism, autophagic cell death has been described, attesting to the complexity of this pathway. Studies have shown that a close interaction exists between autophagy, UPR, ERAD, HSR, and UPS^[91–93]^.

Autophagy maintains quality control of newly synthesized proteins, potentially explaining the high levels of basal autophagy in MM^[94]^. The current consensus is that this process is essential for MM survival as an alternative proteolytic pathway, particularly when other proteostasis pathways are overwhelmed, thus providing a rationale for the combination of autophagy inhibitors with PI in MM^[95]^.

Notably, many agents with proven anti-MM activity, like mTORC1 inhibitor rapamycin and bortezomib itself, were noticed to induce autophagy that thus may represent a potential escape mechanism^[91,96,97]^.

Preclinical studies have shown that p62 contributes to protein homeostasis in MM cells by clearance of redundant misfolded proteins. Importantly, p62 is increased after PI treatment, suggesting a role as an escape mechanism and potential mechanism of resistance to PI^[95]^. Consistently, knocking out p62 increases sensitivity to PI, suggesting this may be a novel, attractive molecular target to overcome PI resistance in MM.

Other mechanisms of bortezomib resistance, in the context of autophagy, include the upregulation of a cytoskeleton protein, profilin-1, which promotes autophagy by binding Beclin-1 complex^[98]^.

Clinical translation of these findings has been attempted by pharmacologic inhibition of this pathway with 3-methyladenine and chloroquine [Figure 2C]^[96]^. Chloroquine inhibits autolysosome-mediated proteolysis by alkalinizing the lysosomal pH. Preclinical studies showed that chloroquine potentiated carfilzomib cytotoxicity and was able to overcome carfilzomib resistance *in vitro*^[99]^. In a phase II clinical trial, the combination of chloroquine and bortezomib/cyclophosphamide showed a modest 14% ORR in patients with refractory myeloma who progressed on a combination of bortezomib and cyclophosphamide^[100]^.

Importantly, given the complexity of the autophagic pathway as well as non-selective targeting of autophagy, combined inhibition of autophagy and proteasome system in preclinical studies yielded conflicting and highly variable results, ranging from synergism to antagonism^[94]^. For instance, a specific study reported that the autophagy inhibitors 3-methyladenine and chloroquine had antagonistic effects when used in combination with bortezomib^[91]^. One potential explanation for these conflicting results is that autophagic cell death can contribute to the anti-MM effects of several agents, including bortezomib, thus representing a double-edge sword.

## ER STRESS AND UNFOLDED PROTEIN RESPONSE

The UPR is a highly conserved cell response to stress, elicited by the accumulation of unfolded proteins in the ER^[11]^. The primary goal of the UPR is to maintain protein homeostasis by specifically halting *de novo* protein synthesis at the level of translation, while selectively inducing the transcription of chaperone molecules to aid in protein folding. The UPR integrates with the UPS via the ERAD in an attempt to resolve proteotoxicity via protein degradation. If homeostasis cannot be achieved, prolonged UPR activation ultimately leads to apoptosis, outlining the double-edged nature of this stress response pathway^[101]^.

The UPR is a tripartite system relying on three distinct stress sensors: inositol-requiring enzyme 1 (IRE1), PERK, and activating transcription factor 6 (ATF6)^[11]^. The three branches of the UPR operate in parallel as feedback loops that mitigate ER stress. Proof-of-principle studies have shown that ER stressors, such as tunicamycin, thapsigargin, and brefeldin A, potentially synergize with PI *in vitro* [Figure 2D]^[14]^. However, the clinical translation of these drugs is limited by the narrow therapeutic index and potential organ toxicity.

Activated IRE1 functions as an endonuclease, resulting in the mRNA splicing and activation of the transcription factor X-box binding protein (XBP1)^[102]^. Together with ATF6, spliced XBP1 (sXBP1) induces lipid biogenesis to sustain ER expansion, chaperone proteins to support nascent ER protein folding, or alternatively initiates ERAD to reduce ER stress^[40]^. High sXBP1 expression in primary MM cells was shown to correlate with poor overall survival, and expression of sXBP1 in B cells reproduces MM phenotype in mice, suggesting that sustained IRE1-XBP1 activation may contribute to MM pathogenesis^[103]^. Recently, decreased XBP1 splicing was shown to associate with de-differentiation from plasma cells to plasmablasts alongside decreased immunoglobulin production, decreased proteasome load, and reduced sensitivity to PI^[14,104]^. MKC-3946, a specific IRE1 endoribonuclease inhibitor, demonstrated substantial anti-MM activity alone or in combination with PI in preclinical studies^[105,106]^. By inhibiting the IRE1 branch of the UPR, MKC-3946 resulted in activation of PERK, downstream eIF2α phosphorylation, ATF4 cleavage and CHOP expression, thereby leading to a terminal UPR and apoptosis^[107]^.

PERK phosphorylates eIF2α, resulting in repression of global protein synthesis, however, if the stress is persistent over time, PERK selectively cleaves and activates the transcription factor ATF4, resulting in the expression of the pro-apoptotic protein CHOP^[2]^. The combination of the selective PERK inhibitor GSK2606414 with bortezomib resulted in synergistic anti-MM activity in preclinical models^[108]^.

Most recently, the HIV protease inhibitor nelfinavir was shown to block ER protein export, triggering a terminal UPR and consequent apoptosis in preclinical MM models, including bortezomib-resistant cell lines^[109,110]^. Importantly, the combination of nelfinavir with bortezomib and dexamethasone showed an impressive ORR of 65% in patients with lenalidomide resistant, bortezomib refractory MM in a phase II, single-arm study^[111,112]^.

Targeting ERAD directly has recently emerged as a way to disrupt intracellular protein metabolism within MM cells. Of note, VCP/p97 is a cytosolic AAA-ATPase necessary for retro-translocation of misfolded proteins from the ER to undergo proteasome-mediated proteolysis as part of ERAD^[113]^. Preclinical studies on CB-5083 have shown robust activity against myeloma cell lines and a number of *in vivo* MM models. Anti-MM synergistic activity of CB-5083 and PI is likely explained by the p97-dependent retro-translocation of the transcription factor NRF1, which transcribes proteasome subunit genes following proteasome activity insufficiency^[114]^. Minor toxicity was observed in untransformed, non-secretory control cells^[114]^. However, a phase I clinical trial of p97 inhibitor CB-5083 was arrested due to off-target effects and retinal toxicities^[115]^.

Finally, PAT-SM6 is a fully human immunoglobulin antibody targeting glucose-regulated protein 78 (GRP78)^[116]^. GRP78 is a major ER chaperone that facilitates protein assembly and regulates ER stress signaling^[117,118]^. PAT-SM6 treatment induces cytotoxicity of MM cells through induction of apoptosis as the main mechanism of action and activation of complement-dependent cytotoxicity as a second hypothesized mechanism. Further, GRP78 is an interesting target in MM due to its sensor function in the UPR activation. PAT-SM6 showed modest clinical activity as a single agent in RRMM, with 33% of patients enrolled in phase Ib trial reaching stable disease^[119]^. Further trials exploring the combination with existing myeloma drugs are planned.

## HEAT SHOCK CHAPERONE PROTEINS

Heat shock proteins (HSP) are molecular chaperones that play a key role in *de novo* protein synthesis, protein folding, multiprotein complex assembly, and protein sorting^[120]^. HSP70 and HSP90 participate in chaperone-mediated autophagy, and they support the redirection of misfolded proteins for prompt degradation^[121–123]^. Compared to normal cells, MM cells are dependent on the HSP chaperone machinery because of the excessive load of misfolded proteins and high levels of DRiPs production^[124]^. HSPs, therefore, help alleviate proteotoxic stress, prevent terminal UPR, and support MM survival^[124]^.

Among HSP, HSP70 and HSP90 are promising therapeutic targets and the two most widely studied HSPs in cancer, with a central role in supporting the folding of proto-oncogenes^[125]^. Specifically, both these proteins have been found to stabilize mutant p53 in an inactive form, thereby allowing cancer cells to evade anti-growth signals^[126]^. In preclinical MM models, HSP70 and HSP90 inhibition result in UPR activation and apoptosis [Figure 2E]^[127–129]^. A combination of HSP90 inhibitors 17-AAG, KW-2478, and retaspimycin with bortezomib showed synergistic MM killing *in vitro*^[130–132]^. Several other HSP90 inhibitors such as NVP-HSP990, PU-H71, SNX5422, and NVP-AUY922 have been tested and showed promising preclinical results in MM^[133–136]^. Although many of these HSP90 inhibitors have completed phase I clinical trial, the narrow therapeutic index and modest clinical significance have hampered further clinical use^[137,138]^. When tested as monotherapy in phase I clinical trial, only Retaspimycin showed modest anti-MM activity, suggesting that a deeper understanding is necessary to overcome drug resistance in a clinical scenario^[139]^. Tanespimycin (17-AAG, KOS-953), an HSP90 small molecule inhibitor, proved effective against MM *in vitro* and has shown encouraging results in phase I/II clinical trials in combination with bortezomib^[140–142]^.

HSP70 inhibitors, such as PER-16, Ver-155008, MAL3-101, have been developed as an alternative therapeutic strategy to HSP90 inhibitors with encouraging preclinical activity^[128,129,136,143]^.

Finally, HSF1, the “master regulator” of the heat shock response, controlling expression of both HSP90 and HSP70, has been investigated as a potential therapeutic target^[124]^. In preclinical studies, several HSF1 inhibitors (e.g., CCT251236, KRIBB11) were found to induce MM cytotoxicity, with associated induction of the UPR^[144]^.

## CONCLUSION

Over the past twenty years, major progress has been made in understanding MM biology. A pathognomonic hallmark of MM is the intense synthesis of Ig, coupled with insufficient proteolytic mechanisms, resulting in pervasive, baseline proteotoxic stress. This intrinsic vulnerability makes MM cells the ideal target of novel therapies designed to disrupt protein synthesis, folding, and degradation. Although disrupting proteostasis via PI has been successful in MM, resistance is inevitable in most patients over time. In this review, we have described potential therapeutic avenues to overcome PI resistance by targeting the protein homeostasis network. Biology-based, clinical use of these agents holds promise to help overcome PI resistance in MM, with the goal of achieving prolonged remission and functional cure for most MM patients [Table 1].

Only through a deep understanding of the fundamental mechanisms of protein homeostasis, novel targets can be identified to overcome PI resistance and improve patient outcome, resulting in long-term control, if not cure, for most MM patients.

## Figures and Tables

**Table 1.** Investigational agents targeting protein homeostasis in MM. This table outlines promising agents targeting protein homeostasis in advanced preclinical or early clinical development in MM, in different phases of a clinical trial

Drug name	Target molecule/mechanism of action	Status	Study design	Clinical trial identifier/PMID
*Proteasome inhibitors*				
Marizomib (NPI-0052)	• Targets all three proteasomal subunits • PolyUb protein accumulation • Caspase 8- and 9-mediated apoptosis	Phase I completed	Marizomib + POM + DEX in RRMM	NCT02103335
		Phase II completed	Marizomib alone in RRMM	NCT00461045
		Phase I completed	Marizomib + Vorinostat in RRMM	NCT00667082
		Phase I completed	Marizomib + DEX	NCT02103335
Oprozomib (ONX 0912, PR-047)	• Inhibits PSMB5 (proteasome), LMP7 (immunoproteasome) • PolyUb protein accumulation and terminal UPR induction • Caspase 8- and 9-mediated apoptosis • p53 and p21 upregulation • miR33b upregulation • PIM1 downregulation • Anti-angiogenesis	Phase Ib/II completed	Oprozomib alone	NCT01416428
		Phase I/II completed	Oprozomib + DEX + LEN/CPM in NDMM	NCT01881789
		Phase Ib/II terminated	Oprozomib + DEX in RRMM	NCT01832727
		Phase I/II completed	Oprozomib + POM + DEX in RRMM	NCT01999335
		Phase I active	Oprozomib IR or GR formulations + DEX + POM in RRMM	NCT02939183
		Phase Ib/II completed	Oprozomib + Melphalan + Prednisone in NDMM (transplant-ineligible)	NCT02072863
*DUBs and ubiquitin receptors inhibitors*				
VLX1570	• Inhibits proteasome USP14 activity	Phase I/II terminated	VLX1570 + DEX in RRMM	NCT02372240
P5091	• Inhibits proteasome USP7 activity	Preclinical	N/A	PMID: 22975377^[58]^
B-AP15	• Blocks USP14 and UCHL5 • Growth arrest via downregulation of CDC25C, CDC2, and cyclin B1 • Induction of caspase-dependent apoptosis • Activation of UPR	Preclinical	N/A	PMID: 24319254^[145]^
RA190	• Inhibits RPN13 and UCHL37	Preclinical	N/A	PMID: 27118409^[60]^
XL177A	• Inhibits USP7	Preclinical	N/A	PMID: 32210275^[67]^
*HDAC inhibitors*				
Ricolinostat (ACY-1215)	• Inhibits HDAC6 • Caspase 8 and 9 mediated apoptosis • PolyUb protein accumulation and terminal UPR induction • Aggresome disruption	Phase Ib/II active	Ricolinostat + POM + DEX in RRMM	NCT02400242
		Phase I/II active	Ricolinostat + LEN + DEX in RRMM	NCT01583283
		Phase I/II completed	Ricolinostat + BTZ + DEX in RRMM	NCT01323751
Citarinostat (ACY-241)	• Inhibits HDAC6 • Downregulation of MYC and IRF4 • Aggresome disruption	Phase I	Citarinostat + POM + DEX in RRMM	NCT02400242
*Autophagy inhibitors*				
Chloroquine	• Inhibits autophagy alkalinizing the lysosomal pH and inhibiting autophagosome and lysosome fusion	Phase I	Chloroquine + BTZ + CPM in RRMM	NCT01438177
3-MA	• Inhibits autophagy at the level of PI3K Class III	Preclinical	N/A	PMID: 19648108^[146]^
Bafilomycin A1	• Inhibits autophagosome and lysosome fusion	Preclinical	N/A	PMID: 21174067^[93]^
*Unfolded protein response modulators*					
Nelfinavir	• Triggers pro-apoptotic PERK pathway • Inhibition of AKT phosphorylation	Phase I/II active	Nelfinavir + LEN + DEX in progressive MM	NCT01555281
		Phase I active	Nelfinavir + BTZ + Metformin in RRMM	NCT03829020
		Phase II completed	Nelfinavir + BTX + DEX in refractory MM	NCT02188537
Sunitinib	• Inhibition of IRE1 activity	Phase II	Sunitinib malate in relapsed MM	NCT00514137
Lovastatin, zolendronic acid, digeranyl bisphosphonate	• Inhibition of isoprenoid biosynthetic pathway and Rab geranyl • Ig light chain accumulation in the ER - activation of UPR	Preclinical	N/A	PMID: 20828814^[147]^
4μ8C	• Inhibition of XBP1 mRNA splicing	Preclinical	N/A	PMID: 22315414^[148]^
MAL3-101	• Induction of XBP1 mRNA splicing following inhibition of HSP70	Preclinical	N/A	PMID: 22750096^[149]^
MKC-3946	• Inhibition of XBP1 mRNA splicing	Preclinical	N/A	PMID: 14559994^[40]^
STF-083010	• Inhibition of XBP1 mRNA splicing	Preclinical	N/A	PMID: 21081713^[106]^
GSK2656157	• Inhibition of PERK and eIF2α phosphorylation, ATF4 translation, and CHOP mRNA expression	Preclinical	N/A	PMID: 23333938^[150]^
CB-5083	• p97 inhibition - polyUb protein accumulation - UPR induction and apoptosis	Phase I terminated	CB-5083 + DEX	NCT02243917
PAT-SM6	• Inhibition of GRP78-UPR induction • Complement-dependent cytotoxicity	Phase I completed	PAT-SM6 single agent in RRMM	NCT01727778
*Heat Shock Protein (HSP) Inhibitors*				
KW-2478	• HSP90 inhibitor • Apoptosis	Phase II completed	KW-2478 + BTZ in RRMM	NCT01063907
NVP-AUY922	• HSP90 inhibitor • Apoptosis • Downregulation of survival pathways	Phase I/II completed	NVP-AUY922 +/− BTZ +/− DEX in RRMM	NCT00708292
IPI-504	• HSP90 inhibitor • Inhibition of UPR	Phase I completed	IPI-504 in RRMM	NCT00113204
Tanespimycin (17-AAG, KOS-953)	• HSP90 inhibitor • Inhibition of downstream signaling pathways • Induction of UPR	Phase II/III completed	Tanespimycin + BTZ in RRMM	NCT00546780
SNX-5422	• HSP90 inhibitor • Apoptosis	Phase I completed	SNX-5422 in RRMM	NCT00595686
NVP-HSP990	• HSP90 inhibitor • Apoptosis • Cell cycle arrest	Preclinical	N/A	PMID: 22309072^[133]^
NVP-BEP800	• HSP90 inhibitor • Apoptosis • Inhibition of STAT3, ERK, AKT pathways	Preclinical	N/A	PMID: 19686236^[151]^
SNX-2112	• HSP90 inhibitor • Cell cycle arrest and cytotoxicity • Inhibition of ERK, AKT pathways • Inhibition of angiogenesis and osteoclastogenesis	Preclinical	N/A	PMID: 18948577^[152]^
MAL3-101	• Inhibition of HSP70 • Induction of XBP1 mRNA splicing • Apoptosis and cell cycle arrest	Preclinical	N/A	PMID: 22750096^[149]^
PU-H71	• Inhibition of HSP90 • Apoptosis and cell cycle arrest, UPR and apoptosis	Preclinical	N/A	PMID: 20977755^[134]^
TAS-116	• HSP90 inhibitor • Induction of apoptosis • Disruption of ERK, AKT pathways	Preclinical	N/A	PMID: 25306900^[153]^
*Selective degradors*				
Phthalimide conjugated degraders	• Bind to CRBN E3 complex on one hand and to specific protein targets on the other to elicit proteasome-mediated degradation	Preclinical	N/A	PMID: 25999370^[154]^

## Data Availability

Not applicable.
